# Molecular epidemiology of Panton-Valentine Leukocidin-positive community-acquired methicillin resistant *Staphylococcus aureus* isolates in pastoral communities of rural south western Uganda

**DOI:** 10.1186/s12879-016-2124-8

**Published:** 2017-01-05

**Authors:** Benon B. Asiimwe, Rossella Baldan, Alberto Trovato, Daniela M. Cirillo

**Affiliations:** 1 Emerging Bacterial Pathogens Unit, IRCCS San Raffaele Scientific Institute, Via Olgettina 58, Milano, Italy; 2 Universita Vita-Salute San Raffaele, Via Olgettina 58, Milano, Italy; 3 Department of Medical Microbiology, Makerere University College of Health Sciences, P. O. Box 7072, Kampala, Uganda

## Abstract

**Background:**

The emergence of multidrug resistant *Staphylococcus aureus* strains, including methicillin resistant (MRSA), is a global concern. Treatment of bacterial infections in Uganda’s health care settings is largely empirical, rarely accompanied by laboratory confirmation. Here we show the burden, characteristics of MRSA and epidemiology of Panton-Valentine Leukocidin (PVL) positive strains in asymptomatic carriers in pastoral households of south-west Uganda.

**Methods:**

Nasal swabs from 253 participants were cultured following standard methodology. MRSA strains were identified by detection of the *mecA* gene and SCC*mec* typing, and PVL genes detected by PCR. Pulsed Field Gel Electrophoresis (PFGE) was done to evaluate possible transmission patterns. *Spa* typing of PVL positive isolates was done to study the epidemiology of virulent strains in this setting.

**Results:**

*S. aureus* was isolated in 29% (*n* = 73) of the participants, of which 48 were MRSA by *mecA* typing. PVL-encoding genes were found in 49.3% (*n* = 36) of the 73 isolates, of which 25 were also *mecA* positive. Among the PVL negative strains (*n* = 37), 62.2% (*n* = 23) carried the *mecA* gene. The most common SCC*me*c type was V, detected in 39 (18 PVL positive and 21 PVL negative) isolates. PFGE clustered 21/36 (58.3%) PVL positive isolates divided in four pulsotypes and 18/37 (48.6%) PVL negative isolates divided in eight pulsotypes. The most prevalent *Spa* types were t318 (26.5%, *n* = 9) and t645 (20.6%, *n* = 7); while other common *Spa* types were t11656 (*n* = 3), t127 (*n* = 3) and t355 (*n* = 3).

**Conclusion:**

The study shows a high prevalence of community acquired (CA)-MRSA, and PVL-positive isolates with two predominant *spa* types in rural Uganda, further complicating infection control strategies in these underprivileged communities.

## Background

About a third of healthy individuals in the community are asymptomatically colonized with *Staphylococcus aures (S. aureus*) in the nostrils [1], a very important finding considering the fact that nasal carriage of *S. aureus* has been associated with subsequent infection [2], and carriers are an important source of spread of infection in communities. A major concern is the world-wide emergence of methicillin resistant *S. aureus* (MRSA) in the community [3]. In contrast with health care associated MRSA (HA-MRSA) infections, community associated MRSA (CA-MRSA) infections can occur in healthy individuals [4], suggesting that these strains have greater virulence. Skin and soft-tissue infections represent about 90% of cases of CA-MRSA infection, mostly characterized by abscesses or cellulitis with purulent drainage [3].

A predominant feature of CA-MRSA is the presence of the Panton-Valentine Leukocidin (*PVL*) genes that encode a *S. aureus* exotoxin that induces lysis of monocytes and neutrophil granulocytes [5]. Additionally, there is evidence that PVL-positive *S. aureus* strains susceptible to methicillin (MSSA) may be reservoirs for the development of PVL-positive MRSA via the integration of the staphylococcal cassette chromosome *mec* (SCC*mec*) elements including the *mecA* gene conferring methicillin resistance [6]. Another key feature of CA-MRSA is that the strains mainly harbor SCC*mec* types IV and V [7–9], and a relationship between CA-MRSA, SCC*mec* type IV and V and PVL has been confirmed in some studies [10, 11].

While efforts have been made to map out the geographical predominance and spread of different CA-MRSA clones worldwide [3], little is known about its magnitude and genetic composition in developing countries, especially in Africa. Molecular analytical techniques such as pulsed field gel electrophoresis (PFGE), multilocus sequence typing (MLST), *spa* and SCC*mec* typing have been used to show both the spread and evolution of MRSA. PFGE is still considered the gold standard for typing MRSA isolates, and is one of the most discriminative typing methods [12]. While MLST is an excellent tool for investigating clonal evolution of MRSA, it is rather expensive, labour intensive and time consuming. Sequences from the polymorphic region of the *S. aureus* protein A (*spa*) gene have been used to develop a single-locus sequence typing technique for MRSA [13] with a discriminatory power between PFGE and MLST, and ability to investigate both molecular evolution and outbreak situations [14], while remaining simple.

The resistance of *S. aureus* to methicillin is caused by the *mecA* gene, located on a mobile genetic element, the staphylococcal cassette chromosome *mec* (SCC*mec*) [7]. Currently, eleven main SCC*mec* types (I to XI) are known [15], and Zhang et al. developed a multiplex PCR for the characterization of SCC*mec* type I to V [16].

In African countries, CA-MRSA has been reported in a few studies in Mali [17], Nigeria [18], Algeria [19], Egypt [20] and Gabon [21]. In Uganda, a few studies have been done at tertiary health care centers [22–25], and these have focused on HA-MRSA. Community studies have focused on *S. aureus* carriage in raw milk and its products as well as in urban milk vendors [26, 27]. We set out to establish the prevalence and molecular epidemiology of *PVL*-positive CA-MRSA isolates from pastoral communities of rural south-western Uganda so as to inform public health on how to develop effective strategies to prevent spread MRSA in these and surrounding communities.

## Methods

### Study area

The study was carried out in rural pastoral households of Kiruhura district, South Western Uganda. The area is a rangeland, populated by agro-pastoralists and seasonally itinerant semi-nomads whose livelihoods mainly depend on consumption and trade of cattle and their products; and small subsistent crop farming.

### Study design and sampling

A cross-sectional study of *S. aureus* carriage in the area was conducted as part of a zoonotic diseases study among pastoral communities of Kiruhura district, South Western Uganda, in 2013. A total of 196 homesteads from two sub-counties (100 from Kanyaryeru and 96 from Sanga) were recruited and sampled. The sub-counties were purposively selected by the zoonoses study because they bordered Lake Mburo National Park, with a porous human-animal interface. All participants did not have a history of recent hospitalization, and the only demographics characteristics recorded were sex and self-reported age. In each household, only a nasal swab was taken for any participant and these were inoculated into Brain-Heart Infusion (BHI) broth in 15 ml propylene tubes and stored on ice before same day transportation to the Department of Microbiology at Mbarara University of Science and Technology for culture.

### Sample culture, isolation and identification

Initial bacterial culture and isolation was done according to methods described by Cheesbrough [28], with minor modifications. Briefly, 50 μl of culture broth was inoculated on 5% sheep blood agar medium and incubated for 18–20 h at 37 °C. Preliminary identification of the bacteria was carried out based on colony characteristics such as shape, size, color and hemolysis patterns. Isolates suspected of being *S. aureus* were shipped in BHI with 20% glycerol to the Emerging Bacterial Pathogens Unit (EBPU) at San Raffaele Scientific Institute in Milan, Italy, for identification and molecular characterization. At the EBPU, all isolates were sub-cultured on mannitol salt agar (Oxoid Ltd, Hampshire, England), then on blood agar (Becton Dickinson, Heidelberg, Germany), and subjected to the tube coagulase test in rabbit plasma with EDTA (Remel, KS, USA). MRSA strains were identified by detection of the *mecA* gene and SCC*mec* typing.

### DNA extraction, SCC*mec* typing and *PVL* genes detection

Bacterial DNA was prepared from isolated colonies suspended in 500 μl Triton X-100 lysis buffer with 1% Triton and 20 μl of 1 mg/ml lysostaphin, incubated at 37 °C for 1 h, followed by phenol-chloroform extraction. SCC*mec* typing was done by multiplex PCR using primers and protocols for SCC*mec* types and subtypes I, II, III, IVa, IVb, IVc, IVd, and V and the *mecA* gene according to Zhang et al. [16]. The MRSA strain COL (SCC*mec* type I, *mec* gene complex B and *ccr* gene complex 1) was used as positive control. Additionally, amplification of the Panton – Valentine Leucocidin (*PVL*) toxin genes, *lukS-PV* and *lukF-PV,* was performed using the *S. aureus* strain ATCC49775 as positive control, and primers and protocols as described by Lina et al. [29].

### Genotyping of isolates

All isolates were subjected to molecular epidemiological analysis by PFGE after *Sma*I digestion using the CHEF Genomic DNA Plug kit (Bio-Rad) according to a standardized protocol [30]. PFGE was run using a CHEF DRIII system (Bio-Rad). The InfoQuest FP (v5.1) software (Bio-Rad Laboratories) was used to analyze PFGE profiles, according to interpretation criteria described by Tenover et al. [12]. Clustering analysis was achieved using Dice similarity coefficients and the unweighted pair group method with averages (UPGMA) at 1.5% optimization and 1.5% position tolerance. PVL-positive strains were further subjected to spa-typing according to Frenay et al. [13]; and *spa* types were assigned on the Ridom SpaServer (http://spaserver.ridom.de) curated by the SeqNet.org initiative [31].

## Results

### Socio-demographic characteristics and *S. aureus* carriage

The study sampled 196 households, and nasal swabs were taken from 253 participants (172 males and 81 females) with average age of 14 years and a range of 6 to 48 years. Of the samples, 132 (52.2%) were *Staphylococcus spp*, of which only 73 were *S. aureus* (carriage rate of 29%) and further characterized. The 73 isolates were obtained from 48 (65.7%) males and 25 (34.3%) females. The average age of the 73 participants whose isolates were characterized was 13, with a range of 6 to 45 years.

### Prevalence of PVL and *mecA* genes

PVL-encoding genes were found in 49.3% (*n* = 36) of the 73 *S. aureus* isolates, while *mecA* was detected in 65.8% (*n* = 48) of the 73 isolates. The proportion of PVL positive isolates carrying the *mecA* gene was 25/36 (69.4%) (Table 1), while 23/37 (62.2%) of the isolates without the PVL gene possessed the *mecA* (Table 2). Among the PVL positive isolates, 18 of the 25 *mecA* positive isolates were SCC*mec* type V, while the other seven were all SCCmec IVc. Among the PVL negative isolates, 21 of the 23 *mecA* positive isolates were SCC*mec* type V, while the other two were type IVa.

**Table 1:** PFGE profiles, *Spa* types, *mecA*, and SCC*mec* types of PVL positive isolates

Isolate ID	Pulsotype	Spa type	*mec*A	SCCmec
T095Na	A1	NT	+	V
T101Na	A1	NT	+	IVc
T047Na	A1	t127	+	IVc
T070Na	A1	t127	+	IVc
T056N	A2	t318	-	-
T099Nd	A3	t2393	+	IVc
T149Na	A4	t645	+	IVc
T101Ng	A5	t318	+	V
T005Na	B1	t318	+	V
T021Nb	B1	t318	+	V
T021Nc	B1	t318	+	V
T097Na	B1	t318	+	V
T125Na	B1	t318	+	V
T141Nc	B1	t318	+	V
T047Nd	B1	t186	+	V
T156N	B1	t186	+	V
T019N	B2	t186	+	V
T119Nb	C1	t355	+	V
T135Na	C2	t729	-	-
T132Nb	C2	t11656	-	-
T139N	C2	t11656	+	V
T145N	C2	t11656	+	V
T022N	C2	t645	-	-
T041Na	C2	t645	-	-
T099Nb	C2	t645	+	V
T063N	C3	t355	+	V
T053N	D1	t1376	+	IVc
T055N	D1	t1376	+	V
T039Na	E1	t318	+	IVc
T041Nb	E2	t645	+	V
T025N	E3	t2393	+	IVc
T030Nb	F1	t064	+	V
T158Nb	G1	t127	-	-
T087	H1	t064	+	V
T155Nd	H2	t509	-	-
T097Nb	I1	t002	-	-

*ID* identification number of the isolate, *+* positive on PCR amplification, *-* negative on PCR amplification, *NT* not typable

**Table 2:** PFGE profiles, *mecA*, and SCCmec types of PVL negative isolates

Isolate ID	Pulsotype	Spa type	*mec*A	SCCmec
T117Nb	E1	t786	-	-
T119Na	E1	t786	-	-
T092Na	E2	t186	-	-
T058Nb	E4	t7662	-	-
T061Nd	L1	t1236	+	V
T061Nb	L1	t1236	+	V
T059Na	L2	Unknown	+	V
T059Nb	L3	Unknown	+	V
T099Na	L3	Unknown	+	V
T021Nd	L4	Unknown	+	V
T032Nc	F1	t064	-	-
T131Nb	F1	t064	-	-
T032Nb	F1	T064	-	-
T061Nc	N1	t2771	+	V
T074Nc	N1	t2771	+	V
T074Nb	N1	t2771	+	V
T029Nb	H1	t002	-	-
T101Nf	H1	t002	+	V
T076N	G1	t5739	+	V
T128Nd	G1	t2680	+	V
T018Nb	G2	t616	+	V
T092Nb	P1	t4353	+	V
T102Nb	P2	t4353	-	-
T127Na	Q1	Unknown	-	-
T137Ng	Q1	t064	-	-
T110Nb	R1	t4523	-	-
T111Na	R2	t5187	+	V
T027Nb	R3	t5187	+	V
T004Nb	Unique	t3772	+	V
T033Nb	Unique	Unknown	+	V
T005Nb	Unique	t951	+	V
T114Ng	Unique	Unknown	-	-
T151Nb	Unique	t5739	+	V
T115Na	Unique	Unknown	+	IVa
T120N	Unique	t5187	+	V
T126N	Unique	NT	+	IVa
T104Nb	Unique	Unknown	-	-

*ID* identification number of the isolate

### Molecular epidemiology of MRSA PVL positive and PVL negative strains

PFGE profiles of PVL positive and negative isolates were analyzed separately to understand any difference in transmission within the two groups. Among the PVL positive isolates, 21/36 (58.3%) strains were found in four clusters (A1, B1, C2, D1, Table 1); while in PVL negative isolates, 18/37 (48.6%) were found in eight clusters (E1, L1, L3, F1, N1, H1, G1, Q1, Table 2). Some family members carried identical strains. In order to relate the genotypes of the isolates in this study to global epidemiology of *S. aureus*, we performed *Spa* typing of the PVL positive isolates. Only two of the 36 isolates could not be typed by this technique, but they belonged to the same pulsotype (A1). In all, there were 13 *Spa* types among the 34 typable PVL positive *S. aureus* isolates. The most prevalent *Spa* types were t318 comprising of 26.5% (*n* = 9), of which seven isolates carried the *mecA* gene; and t645 (20.6%, *n* = 7). The other common *Spa* types were and t11656 (8.8%, *n* = 3) and t127 (*n* = 3). The other *Spa* types, their frequencies and PFGE profiles can be seen in Table 1.

## Discussion

Knowledge of the epidemiology of bacterial infections is very important for appropriate decision-making in the treatment of arising infections. At a community level, it is also important to curb the spread of infection, including multidrug resistant strains. To our knowledge, this is the first investigation of Panton-Valentine Leukocidin-Positive CA-MRSA in asymptomatic semi-nomadic pastoralist communities in the East African region. The main findings of the study are a high prevalence of MRSA and PVL-positive isolates with a predominant *spa* type (t318).

This study isolated 132 Staphylococci isolates from the nares of 253 participants, of which 73 were coagulase positive *S. aureus* (carriage rate of 29%). The 73 isolates of *S. aureus* in our study were obtained from 48 male and 25 female participants, with an average age of 13 years, with 56/73 (76. 7%) of the participants being between 7 and 15 years old. This is similar to observations elsewhere that CA-MRSA infections tend to occur in previously healthy younger patients [21, 32, 33]. Our finding is of public health importance because this school-going age group has potential of disseminating the strains far and wide in the communities. The carriage rate in our study is more than double that in a study on milk men in and around Kampala city, Uganda, where only 11 Staphylococci were isolated from 31 individuals, of which only 4/31 (13%) were *S. aureus* [26]. Our finding, however, is in agreement with statistics from literature suggesting that between 25 to 35% of healthy individuals are asymptomatically colonized with *S. aureus* in the nostrils [1, 21, 34, 35].

In the current study, the proportion of *S. aureus* isolates carrying the *mecA* gene, hence MRSA, was 48/73 (65.8%). This is generally high in comparison to community studies elsewhere. In the urban and peri-urban Kampala, the four isolates from milk men were all MSSA. In a study of indigenous remote African Babongo pygmies living in Waka National Parc, Central Gabon, all 34 *S. aureus* isolates were susceptible to Oxacillin/Methicillin, and did not amplify for the *mecA* gene by PCR. The authors hypothesized that the result could be due to limited use of antibiotics in that population. Studies in North America and Australia, however, have shown that native and indigenous populations have been associated with a high risk of colonization and infection with CA-MRSA which may be related to many of these groups being disadvantaged, due to their association with low socio-economic status, crowded living conditions and frequent use of antibiotics [36, 37]. There have been reports of absolute resistance to penicillin and high percentage resistance to other antibiotics in milk from similar settings in central Uganda, hence risk of spread to humans through the food chain [27]. In our study community there is frequent usage, by farmers, of veterinary antibiotics to treat nearly all ailments in their livestock, due to poor outreach services by the veterinary department. It is therefore more likely that constant contact with antibiotics for animal use, as well as consumption of raw milk and its products without observing drug withdrawal periods, as is the culture in this setting, are limiting future options for the management of multidrug resistant microorganisms in both humans and animals in pastoral communities of Uganda. Among the 48 isolates carrying the *mecA* gene, 39 (81.3%) were type V, the other being type IV or its subtypes (Tables 1 and 2). Type IV and V SCC*mec* elements have strongly been associated with stains causing MRSA infections in persons with no history of hospitalization, hence thought to be more related to CA-MRSA [38]. Furthermore, it has been shown that children may be at a higher risk of infection with SCC*mec* types IV and V, as well as PVL carrying strains, compared to adults [38, 39]. SCC*mec* type V, which comprised of 81.3% of the *mecA* positive strains in our study, is known to be rare in Europe and the United States [33], and only recently seen in Greece [40]. Because SCC*mec* type IV and V are known to be small and highly mobile elements, their dissemination in a community population may be most commonly by transfers of strains from carriers to other individuals or from MRSA strains to Methicillin Sensitive *Staphylococcus aureus* (MSSA strains), or even from coagulase-negative staphylococci strain to an MSSA strain [41]. However, a recent study on patients with surgical site infections (SSI) at the National Referral hospital in Mulago, Kampala, showed that SCC*mec* type V was the most predominant type [22], suggesting the presence of mixed CA-MRSA and HA-MRSA genotypes in hospital settings in Uganda.

A further characterization of the 36 isolates carrying the PVL gene in this study was done. SCC*mec*IV and PVL are known to be molecular markers associated with the emergence of CA-MRSA worldwide [10]. The proportion of strains carrying both the PVL and *mecA* genes was 25/36 (69.4%), while the *mecA* gene was detected in 23/37 (62.2%) of the isolates without the PVL gene. While there was no significant difference in *mecA* carriage, PFGE profiles for both PVL positive and negative isolates show that there were bigger clusters in PVL positive strains (Table 1) compared to PVL negative strains (Table 2). In fact, 18/37 (48.6%) of the PVL negative isolates were in clusters (Table 1) compared to 21/36 (58.3%) of PVL positive isolates, with the largest (B1) comprising of eight isolates (Table 1). These results suggest that PVL positive isolates in the study were more likely to be involved in chains of transmission compared to the PVL negative isolates. There has been recent evidence of familial spread of PVL carrying MSSA strains in Israel [42] and MRSA strains in Italy [43], while a previous study in Greece revealed that a unique clone of PVL-positive MRSA had spread in both the community and hospital settings, and was replacing older clonal types [44]. In Central Gabon, Africa, PVL-encoding genes were detected in 55.9% of study isolates, with authors concluding that the pygmies in that study faced a risk of developing necrotizing infections, due to the virulence characteristic of the PVL. The finding of a high proportion of isolates carrying both the PVL and *mecA* genes in our study may have considerable implications on future strategies for infection control in these underprivileged communities.

Among the PVL positive isolates, there was a predominant circulating *Spa* type, t318, comprising of nine of the 34 (26.5%) typable isolates. This is the first time the *Spa* type has been identified in a Ugandan setting. Moreover, seven strains (77.8%) of this *Spa* type carried the *mecA* gene, pointing to the presence of a potentially virulent methicillin resistant strain circulating in the community. This strain has been found to be pandemic, and mostly PVL positive, also denoted ST30 by multilocus sequence typing. It has recently been mapped to have originated from Australia and disseminated to Brazil, United States, South Africa and Western Europe [3]. However, it is not related to the USA300, belonging to ST8 and leading cause of CA-MRSA in the USA. It has been revealed that PVL is most frequent in pandemic CA-MRSA strains and certain MSSA lineages, including ST30, appear to be a reservoir of CA-MRSA [6]. Surprisingly, ST30 has also been isolated from Babongo pygmies of Gabon in Africa, who are known to have been separated from other humans over millennia ago [21]. In a study of MRSA in five African cities, only one strain of this type was isolated from Antananarivo, and none from Cassablanca (Moroco), Niamey (Niger), Dakar (Senegal) and Yaoundé (Cameroon). While SCC*mec* type V strains have been isolated from hospital settings in Uganda, none were *Spa* type t318 [22]. However, *Spa* type t645, the second most frequent type in our collection (20.6%), also common in Western Europe and the Middle East (http://spaserver.ridom.de), was found to be the most frequent type isolated from SSI at Mulago National referral hospital [22], supporting the notion that there is a changing epidemiology reflected by community associated SCC*mec* genotypes being now more associated with hospital infections as observed elsewhere [23, 45]. However, it is noteworthy that some spa types of PVL+ *S. aureus* in the present study (t186, t729 and t355) have been previously identified in studies from Africa, and they all share the MLST type ST88 [46].

## Conclusion

The main findings of this study are a high prevalence of CA-MRSA, and PVL-positive isolates with a predominant *spa* type in a rural setting in Uganda. This has implication on future strategies for infection control in these underprivileged communities because of possibility of limited treatment options. Antimicrobial stewardship programs may be necessary in Uganda so as to create awareness and avert possible emergence multidrug resistant microorganisms in the near future.
